# Modelling Maternal Depression: An Agent-Based Model to Examine the Complex Relationship between Relative Income and Depression

**DOI:** 10.3390/ijerph19074208

**Published:** 2022-04-01

**Authors:** Claire Benny, Shelby Yamamoto, Sheila McDonald, Radha Chari, Roman Pabayo

**Affiliations:** 1 Edmonton Clinic Health Academy, School of Public Health, University of Alberta, 11405-87 Ave, Edmonton, AB T6G 1C9, Canada; yamamoto@ualberta.ca (S.Y.); pabayo@ualberta.ca (R.P.); 2 Child Development Centre, University of Calgary, 3820-24 Avenue NW, Calgary, AB T2M 1Z7, Canada; sheila.mcdonald@albertahealthservices.ca; 3 Royal Alexandra Hospital, 10240 Kingsway NW, Edmonton, AB T5H 3V9, Canada; radha.chari@albertahealthservices.ca

**Keywords:** mental health, maternal mental health, income inequality, agent-based modelling

## Abstract

Depression is a major public health concern among expectant mothers in Canada. Income inequality has been linked to depression, so interventions for reducing income inequality may reduce the prevalence of maternal depression. The current study aims to simulate the effects of government transfers and increases to minimum wage on depression in mothers. We used agent-based modelling techniques to identify the predicted effects of income inequality reducing programs on maternal depression. Model parameters were identified using the All Our Families cohort dataset and the existing literature. The mean age of our sample was 30 years. The sample was also predominantly white (78.6%) and had at least some post-secondary education (89.1%). When income was increased by just simulating an increase in minimum wage, the proportion of depressed mothers decreased by 2.9% (*p* < 0.005). Likewise, simulating the Canada Child Benefit resulted in a 5.0% decrease in the prevalence of depression (*p* < 0.001) and Ontario’s Universal Basic Income pilot project resulted in a simulated 5.6% decrease in the prevalence of depression (*p* < 0.001). We also assessed simulated changes to the mother’s social networks. Progressive income policies and increasing social networks are predicted to decrease the probability of depression.

## 1. Introduction

Mental illness is a leading cause of disability in Canada. Specifically, depression is among the most common mental illnesses in Canada [1], affecting approximately 5% of Canadians throughout the lifespan [2]. In Canada, the disease burden of mental illness is 1.5-fold higher than all cancers and more than 7-fold that of all infectious diseases [3]. Over their lifetime, 1 in 5 Canadians experience a mental health illness, such as mood and anxiety disorders [4]; however, there are striking gender disparities in mental health outcomes in Canada. Women of reproductive age and girls are more vulnerable to depression and anxiety [5], with the prevalence of elevated depressive symptoms among mothers of young children being particularly high (17–39%) [6].

Of note, the prevalence of depression in the early postpartum period is reported to be somewhere from 10% to 42% [6,7]. In Canada, nearly one-quarter of recent postpartum mothers reported experiencing symptoms of depression or anxiety [8]. Mothers could be especially susceptible to depression, given the hormonal or physiological changes associated with pregnancy [9,10]. Stress regarding parenthood and finances may also be markedly increased during the postnatal period [11]. In turn, depression in expectant mothers can have detrimental impacts on children [11] and may even affect the development of the child [12].

Recent research suggests that socioeconomic position, social inequities, and the social determinants of health may impact women’s mental health [13,14,15]. The circumstances in which people were born, grow, live, work, and age play an important role in the health and well-being of a population, according to the social determinants of health (SDOH) framework [16]. Several determinants of health contribute to the risk of depression, including age, with younger age being associated with increased risk in comparison to their older counterparts [17]; education, with those having lower education being at greater risk for depression in comparison to those with post-secondary education [18,19]; nativity, with non-recent Canadian immigrants experiencing greater depressive symptoms in comparison to those born outside of Canada [18,20]; and ethnicity, with visible minority groups experiencing higher rates of depression in comparison to white Canadians [21]. Furthermore, these circumstances are shaped by the distribution of money, power, and resources at the global, national, and local levels [16]. Characteristics of the social environment, which incorporate social relationships, socioeconomic conditions, and cultural settings within which groups of individuals and families reside and interact, can influence the mental health of a population [16]. Aspects of the social environment, such as the presence of social networks, social cohesion, social capital, family relationships, and involvement with community activities, have been shown to be associated with mental health outcomes [15]. Income inequality is an important example of a risk factor for health that interacts with our social environments. The ability to access childcare [22] and neighborhood safety and low crime [23] are also associated with a decreased risk of depression. Notably, income inequality is characterized by the gap between the highest and lowest earners within a given residential area. Independent of an individual’s own income level, the distribution of incomes in society has been hypothesized to influence the risk of depression [24] and modelling the change in depression solely due to changes in absolute income has been written about extensively in the academic literature [18,25].

Along with increases in rates of depression in Canada, income inequality has been increasing in major cities [26]. Income inequality is typically measured using the Gini coefficient, where a value of zero represents perfect equality or everyone being measured has the exact same income. A value of one represents perfect inequality, where there is a gap between high- and low-income earners within a given area. While the after-tax income Gini coefficient was reported to be 0.29 in Canada in 2019 [21], the Gini coefficient in Calgary, Alberta was well above average at 0.40 [26]. In fact, Calgary has the third-highest Gini coefficient among all Canadian cities, is the largest city in Alberta, and is the third-most populous city in Canada with a population of 1,306,784 [27].

There are several proposed mechanisms to describe how income inequality affects depression. The relative deprivation mechanisms indicates that neighborhoods with high levels of income inequality might lead to stressful comparisons [28]. These invidious social comparisons might intensify feelings of competition, making one’s status more important compared with societies with more equitable income distributions [28]. Social comparison is an important concept when studying depression as depressed individuals are more likely to rate others as better off than themselves [29,30]. Individuals often compare their income, and other social statuses, to the known or perceived income of those around them. Having an income greater than others in one’s social network can increase happiness while having a perceived income lower than others has been observed to be associated with depression [31,32,33]. Income inequality also erodes social cohesion and trust [34]. Social cohesion is defined as the extent of connectedness and solidarity among groups and individuals in a neighborhood and is linked to depression amongst youth, with individuals in highly unequal neighborhoods more likely to report low social cohesion [35]. Additionally, previous research has shown an association between civic participation, which is an indicator of social cohesion, and depression among women [36,37,38].

Moreover, research has shown links between income inequality and depression, particularly amongst women and girls; for example, researchers have found that adolescent girls living in the most unequal neighborhoods in Boston are more likely to be depressed, but not boys [35]. This may because girls and women react differently to stressful situations resulting from unequal environments, typically exhibiting internalizing behaviours that are more likely to manifest in depressive disorders while boys are more likely to exhibit externalizing behaviours [39,40]. As noted, women of childbearing age are at an increased risk of depression compared to men of similar age [41,42] and women of non-childbearing age [43]. Pregnant women and mothers are also susceptible during the first months postpartum, given hormonal and emotional fluctuations; thus, cases of prenatal depression can often go undiagnosed and untreated [41]. Maternal depression is also especially important because it can have deleterious consequences for child development [12,44].

Existing research demonstrates that income inequality can be reduced via government transfers and subsidy programs [45,46,47], with government transfers being defined by Statistics Canada as all cash benefits received from municipal, provincial, territorial, or federal governments [48]. These transfers can either be targeted to specific population groups or to the general population. In Canada, there are targeted federal programs such as the federal Old Age Security [49] and the Canada Child Benefit (CCB) [50] as well as provincial programs such as the Alberta Child Benefit (ACB) [51,52]. These programs give cash transfers to specific segments of the population, such as seniors or families with young children, at the federal and provincial levels, respectively. There have also been general programs such as MINCOME, a universal basic income program in Manitoba [53], and Ontario’s Universal Basic Income (ON UBI) pilot program [54]. These government transfers can reduce inequality by being progressive, in that low-income households receive more money than high-income households. For instance, under the CCB, benefits are reduced beginning with households with a net income of at least $31,120 [50]; whereas under ACB, benefits are reduced in households with a net income of $26,141 or higher [52]. Additionally, by reducing income inequality and stress associated with financial pressures, such programs have been demonstrated to reduce the prevalence of depression [55] and depressive symptoms [56]. Based on the existing literature, it is unclear whether this is the case for expectant mothers specifically; however, the current study will investigate this gap in the field. Further, given that income inequality is concentrated within urban areas [26], the current study will simulate the effects of programs that reduce income inequality on maternal depression using baseline data from a sample of Calgary, Canada mothers. We hypothesize that government transfers and increases to minimum wage will reduce the risk for depression in these simulated models.

## 2. Methods

### 2.1. Model Overview

Agent-based modelling (ABM) is a systems science modelling technique used to simulate complex systems of individuals, known as agents. In these models, agents can make decisions and interact with other agents [57]. These interactions with other agents can influence decisions made by those agents [57] and those decisions made by the agents can vary from simple ‘if-then’ decisions to more complex decision-making models [58]. According to Macal and North [57], ABMs usually have three components: (1) agents, (2) relationships between agents, and (3) the agents’ environment. Each agent has their own attributes and rules for making decisions. These rules for making decisions include how agents will act in their relationship with other agents. Finally, the agents also have rules for interacting with their environment. In our ABM, our agents are expectant mothers with individual attributes such as household income, age, and education. The agents also have attributes based on their environment, including nativity, ethnicity, and mean household income in their neighborhood. Agents in our models can make decisions whether to increase or reduce their social networks and whether to remove depressed persons from their social networks. Agent-based modelling was necessary for the current study because of the complexity of the simulated relationship between policies and programs that reduce income inequality, social networks, and maternal depression. Allowing agents to make choices about their social networks increases the real-world applicability of the current study.

An initial logistic model based on secondary data was developed to predict whether an expectant mother experienced depression, as indicated by a respondent’s Edinburgh Postnatal Depression Scale score from the All Our Families (AOF) study. Additionally, from the AOF study, the household income and mother’s education level, age, ethnicity, ability to obtain prenatal care, sense of safety in her neighborhood, and sense of the level of crime in her neighborhood were used as individual-level control variables. Additional confounding variables were calculated from the 2006 Census at the dissemination area (DA) level and linked to the AOF cohort data. Dissemination areas are the smallest geographical areas for which Census data are disseminated [59].

In our analysis, we modelled the decreasing relative income differences, as a result from implementing social programs (i.e., minimum wage increases, ON UBI, ACB, and CCB), on depression. Changing an individual’s income causes changes to their relative income and, thus, depressive scores. We modelled the effect of income differences joint with changes to social networks on depression. ABM was the most appropriate method to predict the overall effects when income inequality changes. As Tracy et al. [60] stated, the objectives for ABMs in public health are to predict and explain complex systems. ABM is also appropriate as previous studies have shown ABM results to be robust, even when researchers were required to make assumptions about the model with few parameters to inform the assumptions [61]. Analyses were conducted using Python. 

### 2.2. Sample Characteristics

The data for the basis of our simulation analysis come from the All Our Families (AOF) study, a community-based, cohort study developed to investigate the relationships between the prenatal and early life period outcomes for infants, children, and mothers [62]. Women were recruited both actively (e.g., contacted directly in medical laboratories or in primary care waiting rooms) and passively (e.g., posters and other paper materials) from Calgary, Canada. The first wave of the AOF study was conducted in Calgary, Canada, in 2008 and consisted of 3387 expectant mothers. Neighborhood characteristics were derived from data collected during the 2006 Statistics Canada Census. The 2006 Census was used as it was collected just before the start of the AOF study and is generally considered to be more reliable than the 2011 Census [63,64,65].

The mean age of our sample was 30 years, ranging from 18 to 47 (standard deviation = 4.6). The sample was also predominantly white (78.6%), had at least some university or college education (89.1%), and did not receive any government income support (94.6%). Additional sample characteristics are described in more detail in Table 1.

### 2.3. Interventions

We used four methods to reduce income inequality for the agents. We increased income, thereby reducing income inequality, according to the ACB, the CCB, the ON UBI pilot program, and by increasing minimum wage [51,66,67]. For increasing minimum wage, we assumed only one person in the household worked a minimum wage job, with a standard 40 h per week [68] and 50 paid weeks per year [69]. Initial household income was based on Census data and assuming one, full-time worker appears to be a reasonable assumption in a myriad of combinations of number of income-earners and weekly hours worked. Each of the government programs and minimum wage methods set a minimum household income for each agent and not all households in the simulation were eligible for these programs if their after-tax household income was above the minimum income thresholds. The two-child benefit (ACB and CCB) and the UBI programs also increased the household income of higher-income households but the amount of money each household received diminished as household income increased [52,54,67,70].

For increasing the minimum wage, we followed the range of historical increases in Alberta but increased the minimum wage by the same amount at each step of our simulation. The minimum wage in Alberta was $8.40/h on 1 April 2008, about the time the AOF study data collection began [67]. The minimum wage increased in steps to $15/h on 1 October 2018 [67]. We compared the likelihood of being depressed before and after increasing household income according to the parameters of the child benefit or ON UBI program. As previously discussed, benefits are reduced for those with a net income of at least $31,120 [50] and $26,141 or higher [52] for CCB and ACB, respectively.

### 2.4. Modelling

For each step in our modelling, we increased the household income of the agents with the lowest household incomes (i.e., that below what would be earned by one minimum wage earner) to a minimum household income assuming one person in the household had a full-time, minimum wage job. We used the new household income accounting for each of the four interventions (minimum wage increase, ACB, CCB, and ON UBI) at each step to predict whether an agent continued to be depressed, became depressed, or stopped being depressed. 

Eight simulations were run to evaluate the effects of income changes and social network changes on depression. The models tested eight distinct conditions: (1) increases to income only, (2) increases to income only + baseline presence of social support, (3) increases to income + decreases in depressed persons in the social networks, (4) increases to income + increases in social networks, (5) increases to income + decreases in depressed persons in the social networks + increases in social networks, (6) decreases in depressed persons in the social networks only, (7) decreases in depressed persons in the social networks + increases in social networks, and (8) increases in social networks only. Agents could make decisions on whether they increased their social networks or removed depressed persons from their social networks.

### 2.5. Measures

#### 2.5.1. Exposure

The primary exposure variable in this study was relative income, which was calculated to be the difference between the respondent’s annual household income and the median after-tax household income of the dissemination area (DA) from the 2006 Census. After-tax household income was used to reflect a household’s available income.

#### 2.5.2. Outcome

Depression was measured in the 2008 AOF study using the Edinburgh Postnatal Depression Scale (EPDS), which is a series of Likert scale questions assessing depressive symptoms with questions including, but not limited to: In the past 7 days, “I have been able to laugh and see the funny side of things”, “I have looked forward with enjoyment to things”, “I have blamed myself unnecessarily when things went wrong”, and “I have felt sad or miserable” [71,72]. A higher score on the EPDS would correspond to a higher depression score. The outcome was dichotomized in our study, with a cut-off score of 10 or higher indicating a “case” of depression [73]. The EPDS has been previously validated for use in pre- and postpartum women [73]. 

#### 2.5.3. Covariates

Presence of social support was identified at baseline in the AOF using the question: “How many close friends and/or close relatives do you have that you feel at ease with and can talk to about what is on your mind?” [62,74]. Those indicating they had at least one close friend and/or close relative were understood to have social support present at baseline.

DA-level characteristics were identified in the 2006 Census and included the median after-tax household income, percentage of families in a dissemination area living below the low-income cut-off (LICO), percentage of households spending more than 30% of income on shelter costs, and percentage of recent immigrants. Once the statistical model was created, its coefficients were used to simulate the impact of policy changes on depression in expectant mothers.

## 3. Results

Results for trials of the four methods of increasing household income are shown in Table 2. For each simulation, we averaged 17,865 agents representing 183 Calgary DAs, as based on the AOF cohort. The four methods of decreasing income inequality: raising minimum wage, applying the Alberta Child Benefit (ACB), applying the Canada Child Benefit (CCB), and applying Ontario’s Universal Basic Income (ON UBI). The “*n*” column is the number of agents in the trial who were assigned a valid community and DA. For each trial, the logit model’s coefficients and the agent’s assigned attributes were used to predict whether an agent was depressed. Using the Edinburgh Postnatal Depression Scale score, 34.4% of expectant mothers were depressed. For each trial, we calculated the expected probability that an agent was depressed and selected a probability value such that 34.4% of agents were predicted to be depressed. The number of agents predicted to be depressed at the beginning of each trial is in the “baseline” depressed column and the number of agents predicted to be depressed at the end of each trial is shown in the “follow-up” depressed column.

Minimum wage increases, the CCB and ON UBI programs were predicted to have a statistically significant effect on maternal depression. When income was increased by just simulating an increase in minimum wage, the proportion of depressed mothers decreases by 2.9% (*p* < 0.005). Likewise, the addition of a CCB resulted in a 5.0% decrease in the prevalence of depression (*p* < 0.001) and the addition of ON UBI pilot project resulted in a 5.6% decrease in the prevalence of depression (*p* < 0.001). When income was increased by simulating the addition of the ACB, there were no statistically significant effects on the prevalence of maternal depression. Given that the ACB model is not statistically significant, it is possible that 25 agents predicted to no longer be depressed at the end of the simulation may only have occurred due to random chance.

Table 3 demonstrates the results of the agent-based models accounting for changes to social networks among mothers. For example, in the simulation, an agent’s income was increased, and agents had the choice to remove a depressed friends and/or close relatives from their social network or increase their social networks. Increases in income and presence of social networks was simulated to reduce the prevalence of depression among mothers by 2.1% (*p* = 0.038). Conversely, removing depressed persons from their social network increased the simulated prevalence of depression by 14.7% (*p* < 0.001) and when paired with increases in income, increased the simulated prevalence of depression by 12.8% (*p* < 0.001). Increases in social networks were associated with stark reductions in the simulated prevalence of depression (% change = 43.0%, *p* < 0.001) and when paired with increases in income, reduced the simulated prevalence by 46.1% (*p* < 0.001). When increasing social networks, but removing depressed persons from social networks, the simulated prevalence of depression was reported to be reduced by 20.7% (*p* < 0.001) and with increased income, reduced the simulated prevalence of depression by 22.9% (*p*< 0.001).

## 4. Discussion

We used agent-based modelling techniques to simulate the effect of income inequality reducing interventions on depression and agent-based modelling techniques to simulate the effect income inequality reducing interventions paired with changes to social networks on depression among mothers living in Calgary. As hypothesized, income inequality between the agents was decreased by increasing the income of the agents with the lowest incomes. While it was important to show that raising minimum wage did significantly lower the number of expectant mothers who are depressed, the real-life effect of increasing minimum wage would likely be similar to the government assistance models in more marginalized samples. The models run using the CCB or ON UBI show that an increase in household income for those more disadvantaged would have a greater benefit than increasing minimum wage. The assumptions about the effect of minimum wage effectively set a minimum household income and did not affect households above that minimum income. An actual increase in minimum wage would likely affect households in a similar fashion to the CCB or ON UBI. That is, household income would be increased in a progressive manner such that lower-income households would see more benefit than higher-income households.

According to our modelling, an expectant mother was less likely to be depressed if she had at least one close friend and/or relative. There is, however, a complex risk–reward situation where adding a friend is a benefit, but it is unclear if that benefit might be reduced if persons with depression are added to the agent’s social networks. Our simulations show that, overall, mothers were more likely to be depressed if they removed a depressed friend from their social network. This is surprising considering that most, albeit not all [75], existing evidence has shown that depression is socially contagious [76,77,78]. However, these results ultimately show that increases to social networks are beneficial and reduce the likelihood of a mother having depression, whether or not persons in that social network are depressed. Overall, the findings from our agent-based model support empirical evidence that social support obtained from social networks is related with benefits to mental health, particularly among expectant mothers [79].

The simulations examined in this study are based on real-world interventions across Canada. Child benefit programs have been proven effective for reducing child poverty rates [66,70]; minimum wage increases have been associated with reductions in smoking prevalence [80], depressive symptoms [56], and improvements in population health [80,81,82,83] and earnings [84]; and universal basic income programs have been associated with reductions in psychological stress. These reductions in stress may subsequently reduce conditions such as cardiovascular disease, upper respiratory tract infections, and depression [55,85,86,87]. While not yet evaluated in the existing literature, it stands to reason that such programs could have beneficial effects on depression, specifically among mothers. Aside from the benefit of increasing available income, childcare benefits may also ease the burden of stress of accessing childcare or the affordability of childcare in lower-income mothers, which is noted to be significantly associated with maternal depression in the existing literature [22]. This is important, given the high prevalence of depression in pregnancy and the postpartum period. Indeed, reducing maternal depression can also have beneficial effects for child development and for birth outcomes [12]. This simulation study allows us to predict the effects of income inequality reducing programs on maternal depression and offer insight and inform discussions on such programs.

### Limitations

There are some limitations to our agent-based models. Our research approach is based on the results of a statistical model using expectant mothers. While our results are representative of previous research on depression and inequality [56,88], our results may only be applicable to expectant mothers due to our initial model and assumptions. As such, our results are most applicable to policy changes targeted at expectant mothers, such as the Alberta Child Benefit or Canada Child Benefit. The Governments of Alberta [66] and Canada [50,70] report that those child benefits have decreased child poverty and our simulation demonstrates that the Canada Child Benefit could also reduce the prevalence of maternal depression. In addition, the original risk estimates were unable to account for social mobility or secular trends in income because these constructs were not available. This is important, because income volatility or changes in income independent of government transfers may also impact the risk for depression.

It is important to note that the decision-making capabilities of the agents (e.g., changes in social networks, including increasing the size of an agent’s social network, decreasing the size of an agent’s social network, or removing depressed persons from an agent’s social network) were based on a single-item question in the AOF dataset. While validated measures of social support do exist, such constructs were not available for the current study. Future studies should consider the use of richer measures of social networks and social support in studies on maternal depression.

Additionally, it is possible the lowest-income households may not see the greatest benefit when minimum wage alone is increased. The real increase in household income would be dependent upon how much hourly pay rates were increased to meet the new minimum wage and the number of hours worked. A household where individuals worked many hours for a wage well below the new minimum wage would see much more benefit than a household where individuals worked fewer hours for a wage just below the new minimum wage, all else being equal [89]. The overall effect of increasing minimum wage is complicated and could result in workers working fewer hours, inflation, a reduction in benefits, or a loss of jobs for lower-skilled workers [90]. However, it should be noted that evidence from natural experiments does indeed demonstrate that minimum wage increases have no effect on employment of low-wage workers [91]. Moreover, given that our agents primarily lived in high-income households, it is possible that the effects of these income inequality reducing programs may not be predicted to be as strong as that may be in samples with lower average household incomes. Qualitative research may help to support the current work and contextualize this quantitative study [92]. Future studies should aim to simulate the effects of income inequality reducing programs in lower-income samples. Researchers may also consider evaluating increases to minimum wage on maternal depression using quasi-experimental methods. 

## 5. Conclusions

This analysis provides simulated evidence on how economic and social factors may decrease the burden of mental illness. The results support our hypothesis that government transfers and increases to minimum wage will reduce the risk of depression in these simulated models. Despite this study’s limitations, we provided a key contribution to the academic literature by exploring the potential effects of programs that reduce income inequality on the risk for depression in expectant mothers. The results show that reducing relative income inequality through any means can reduce the possibility of depression in expectant mothers. To improve the mental health of expectant mothers, a combination of progressive income or child benefit programs aimed at reducing inequality and programs to increase social networks for expectant mothers is likely key. Due to the complexity of the impact of economic and social factors on depression, this analysis also shows the advantages of using agent-based modelling. Even though the model used here is supported by previous research and the AOF survey data, future research should be undertaken to improve the decision-making logic of agents in the model. Improving the logic of the ABM would likely reveal more nuance on the interaction of economic and social factors in mental health. The current study can inform policies and programs that reduce income inequality and will bolster support for such interventions with the hopes of mitigating the adverse effect that widening incomes may have on maternal mental health.

## Figures and Tables

**Table 1:** Baseline characteristics of the population.

Variable		Mean (Std Dev)	Range (Min, Max)
Age	Years	30.60 (4.55)	18, 47
		*n*	%
Education	High school or less	367	10.94
	Some or completed university, college	2458	73.29
	Some or completed graduate school	529	15.77
Nativity	Born in Canada	2628	78.26
	Not born in Canada	730	21.74
Ethnicity	White	2636	78.64
	Non-white	716	21.36
Income (annual, before taxes)	$39,999 or less	298	9.17
	$40,000 to $79,999	717	22.06
	$80,000 or more	2235	68.77
Received government income support	Yes	180	5.38
	No	3163	94.62

**Table 2:** Results of agent-based models simulating the effects of four income inequality reducing programs on maternal depression.

Intervention	*n*	Baseline Depressed	Baseline % Depressed	Follow-Up Depressed	Follow-Up % Depressed	% Reduction in the Prevalence of Depression	*p*-Value
Minimum Wage Increase	17,874	6154	34.43%	5974	33.42%	2.92	0.005
Alberta Child Benefit	17,881	6157	34.43%	6132	34.29%	0.41	0.694
Canada Child Benefit	17,866	6151	34.43%	5846	32.72%	4.96	<0.001
Ontario’s Universal Basic Income	17,874	6154	34.43%	5807	32.49%	5.64	<0.001

**Table 3:** Results from the eight agent-based models accounting for simulated changes to social networks.

Model	*n*	Baseline Depressed	Follow-Up Depressed	Incident Cases of Depression over Follow-Up ^a^	Reduced Cases of Depression over Follow-Up ^b^	% Change in the Prevalence of Depression ^c^	*p*-Value
Income only	17,874	6154	5974	0	180	2.92	0.005
Income + presence of social networks	17,873	6154	6022	0	132	2.14	0.038
Income + reduced depressed persons in the social networks	17,882	6157	6944	838	51	−12.78	<0.001
Income + increase in social networks	17,865	6151	3318	0	2833	46.06	<0.001
Income + reduced depressed persons in the social networks + increase in social networks	17,863	6150	4744	24	1430	22.86	<0.001
Reduced depressed persons in the social networks	17,879	6156	7062	906	0	−14.72	<0.001
Reduced depressed persons in the social networks + increase in social networks	17,881	6157	4880	18	1295	20.74	<0.001
Increase in social networks	17,862	6150	3508	0	2642	42.96	<0.001

^a^ Number of new cases of depression predicted by the simulation; ^b^ number of cases of depression reduced from baseline predicted by the simulation; ^c^ % change in the prevalence of depression from baseline to follow-up predicted by the simulation.

## Data Availability

Data from the All Our Families cohort are available through https://allourfamiliesstudy.com/data-access/ (accessed on 28 March 2022).
